# Non-contrast Magnetic Resonance Urography Versus Computed Tomography Urography for the Evaluation of Obstructive Uropathy: A Prospective Paired Comparative Study

**DOI:** 10.7759/cureus.117788

**Published:** 2026-10-11

**Authors:** Sriramula Sai Dilip, Amarapilli Appalaraju, Immadi Chandrasekhar, Layeeq Fatima

**Affiliations:** 1 Urology, Andhra Medical College, Visakhapatnam, IND

**Keywords:** computed tomography urography, magnetic resonance urography (mru), mega ureter, obstructive uropathy, puj obstruction, ureteric calculus, ureteric obstruction

## Abstract

Background

Obstructive uropathy is a common condition that can damage the kidneys if it is not detected and treated early. Appropriate imaging is needed to identify the location and cause of the obstruction. Computed tomography urography (CTU) is widely used for evaluating urinary tract obstruction, while non-contrast magnetic resonance urography (MRU) may provide an alternative when ionizing radiation or iodinated contrast is undesirable.

Aim

To prospectively compare CTU and non-contrast MRU for the accurate identification of the cause and level of obstruction against an intervention-based reference standard in patients with suspected obstructive uropathy.

Materials and methods

This observational study was carried out over two years (January 2023-December 2024) in the Department of Urology, King George Hospital, Visakhapatnam. We prospectively enrolled 30 eligible patients during the study period. Each patient underwent both CTU and non-contrast MRU, allowing a paired within-patient comparison. CTU and MRU were performed within two weeks of each other and were interpreted by different blinded radiologists. The intervention was performed within one week of MRU. The reference standard consisted of intervention findings or histopathology in 29 patients and follow-up in one patient.

Results

Against the reference standard, CTU correctly identified both the cause and level of obstruction in 30 of 30 patients (100%), while MRU correctly identified both in 28 of 30 patients (93.3%). Both modalities identified all 17 calculi, seven strictures, and three urothelial or bladder tumors. Of the three congenital cases (pelviureteric junction (PUJ) obstruction or megaureter), CTU identified all three, while MRU identified one.

Conclusion

In this prospective cohort, non-contrast MRU correctly identified the cause and level of urinary tract obstruction in 28 of 30 patients, compared with 30 of 30 for CTU. The two MRU failures involved congenital abnormalities: primary obstructive megaureter and PUJ obstruction with crossed fused renal ectopia. The study was not designed to test equivalence or non-inferiority of MRU to CTU. Non-contrast MRU could be an alternative for selected patients to avoid radiation or iodinated contrast. Larger multicentre studies with standardized protocols, blinded interpretation, and independent reference standards are needed.

## Introduction

Obstructive uropathy occurs when the flow of urine is blocked somewhere along the urinary tract, either due to a structural or functional problem. If left undetected, it can lead to hydronephrosis and progressive kidney damage, potentially resulting in irreversible renal failure [1]. The common causes include urolithiasis, strictures, congenital anomalies, tumours, and inflammatory conditions [2]. Early and accurate diagnosis is essential both to prevent long-term complications and to plan appropriate treatment.

Imaging is central to the evaluation of these patients. Ultrasound is usually the first imaging test performed, mainly because it is widely available and does not involve radiation. However, its accuracy depends considerably on the operator, and it does not always clearly visualize the entire urinary tract [3]. Intravenous urography (IVU), once the standard investigation, has now largely been replaced by cross-sectional imaging techniques [4].

Computed tomography urography (CTU) has become widely used for evaluating urinary tract obstruction because of its high resolution, rapid acquisition, and ability to assess both the urinary tract and surrounding structures in a single study [5]. It is particularly effective in detecting urinary calculi and urothelial malignancies [6]. Non-contrast CT is the standard imaging test for detecting stones; multiphasic CTU includes an unenhanced phase. However, non-contrast CT was not used as a comparator in the present study. The main drawbacks of CTU are radiation exposure and the use of iodinated contrast, which may pose risks in patients with renal impairment or contrast allergies and during pregnancy [7].

Magnetic resonance urography (MRU), especially in its non-contrast form, is emerging as a useful alternative. It relies heavily on T2-weighted sequences to visualize fluid-filled structures such as the renal pelvis, ureters, and bladder without requiring an external contrast agent [8]. This makes non-contrast MRU well suited for children, pregnant women, and patients with reduced renal function [9]. However, MRU may be less effective than CT for the direct visualization and characterization of small urinary calculi, and the scan takes longer, requiring greater patient cooperation [10,11].

MRU is being used increasingly, especially when CT is contraindicated, but comparative data against CTU in obstructive uropathy are limited. This study compared CTU and non-contrast MRU for the accurate identification of the cause and level of obstruction against an intervention-based reference standard in patients with suspected obstructive uropathy. It was a descriptive comparison and was not designed to test agreement, equivalence, or non-inferiority.

## Materials and methods

Study design and duration

This was a prospective, single-centre, paired comparative observational study conducted in the Department of Urology, King George Hospital, Visakhapatnam, between January 2023 and December 2024.

Ethical approval and informed consent

The study received approval from the Institutional Ethics Committee at Andhra Medical College, Visakhapatnam (approval number: 16/IEC AMC/JAN 2023). All adult participants provided written informed consent before enrolment, and parents or guardians provided consent for children.

Study population

The study included patients with clinically suspected obstructive urinary tract lesions, particularly those in whom USG showed features of urinary tract obstruction but was inconclusive, and who were referred for advanced imaging.

Sampling method and sample size

Eligible patients were enrolled prospectively according to predefined criteria. Thirty patients were enrolled. Each underwent CTU first, followed by non-contrast MRU within two weeks. The interval depended on the patient's clinical condition and MRU slot availability; in severe cases, MRU was performed as early as possible. Upon comparison of the two scans, no change was observed in stone position or degree of hydronephrosis. No patient underwent DJ stent placement or nephrostomy between CTU and MRU or between imaging and intervention. Intervention was performed within one week after MRU (i.e., up to approximately three weeks after CTU) in the 29 patients who underwent intervention. The sample size of 30 was a practical limit determined by imaging resources, and no formal sample-size calculation was performed.

Inclusion criteria

Patients were included if they were referred for CTU with suspected obstructive lesions of the urinary tract and if other radiological investigations had yielded inconclusive results. Patients qualified for inclusion if they met both predefined criteria.

Exclusion criteria

We excluded patients with absolute contraindications to MRI (cardiac pacemakers, intracranial aneurysmal clips, implanted hearing aids, metallic intraocular foreign bodies, or severe claustrophobia); obstructive uropathy due to causes distal to the urinary bladder (for example, prostatic or urethral obstruction); serum creatinine above 1.5 mg/dL (normal range: 0.5-1.5 mg/dL), which was the institutional upper safety limit for the administration of iodinated contrast media for CT urography; the need for urgent decompression; and CT scans performed elsewhere that did not follow the study CTU protocol.

CTU protocol

CTU was performed using a 64-slice multidetector CT scanner (SOMATOM Definition AS+, Siemens, Erlangen, Germany). Images were reconstructed at a slice thickness of 5 mm. The median radiation dose-length product (DLP) was 485 mGy·cm (range: 210-890 mGy·cm). According to our institutional protocol, patients fasted for 4-6 hours and drank 500 mL of water 30-45 minutes before the scan. No diuretics were administered before contrast injection. For the infant and the six-year-old child, the dose was adjusted according to body weight, and sedation was used. A non-ionic iodinated contrast agent (Omnipaque; GE HealthCare, Chicago, IL, USA) was administered at a dose of 1 mL/kg body weight, with a minimum volume of 60 mL. Scans were obtained in an unenhanced (plain) phase to detect calculi, followed by a corticomedullary phase at 20-40 seconds, a nephrogenic phase at 80-90 seconds, an excretory phase at 180-300 seconds, and a delayed phase at 10-15 minutes after contrast injection.

MRU protocol

MRU was conducted on a 1.5-Tesla MRI scanner (MAGNETOM Aera, Siemens Healthineers, Erlangen, Germany) with a phased-array body coil. Coronal and axial HASTE images were captured at 3-4 mm slice thickness, while a 3D SPACE sequence was acquired with a reconstructed voxel size of about 1 mm. The infant and the six-year-old child were sedated by the pediatric anaesthesiology team for motion management. The other patients underwent MRU without sedation. No oral hydration or diuretic was used for MRU. The protocol included coronal T2-weighted Half-Fourier Acquisition Single-shot Turbo Spin Echo (HASTE) with fat suppression; axial T2-weighted HASTE covering the entire abdomen and retroperitoneal space; single-shot turbo spin echo (SSTSE) with an acquisition time of about 7 seconds; and 3D SPACE (Sampling Perfection with Application optimized Contrasts using different flip angle Evolutions) sequences for high-resolution imaging. The field of view was adjusted according to the patient's build. Maximum intensity projection (MIP) images were reconstructed from the 3D datasets for better visualization of the urinary tract. The complete MRU protocol took approximately 25-30 minutes.

Confirmation of diagnosis (reference standard)

In 29 of 30 patients, diagnosis was confirmed at the time of surgical or endourological intervention: ureteroscopic or percutaneous stone retrieval for calculi, endoscopic or open repair for strictures, and pyeloplasty or ureteric reimplantation for congenital lesions. Histopathology confirmed the diagnosis in the three patients with suspected malignancy. We performed diuretic renography in patients with congenital obstruction as part of their routine work-up before surgery. Intervention was done within one week of MRU imaging. For the remaining one patient (with ureteric stricture), who underwent ureteroscopy one year before enrolment and was managed non-surgically during the study period, we established the final diagnosis based on clinical course, repeat ultrasound imaging, and follow-up of at least 12 months. No patient was excluded from analysis for want of a reference diagnosis.

Radiologic interpretation and blinding

One radiologist (M.D. in Radiodiagnosis, 12 years of experience in genitourinary imaging) reported all CTU examinations, and a different radiologist (M.D. in Radiodiagnosis, 19 years of experience) reported all MRU examinations, so no radiologist reported both modalities for the same patient. Both were blinded to the ultrasound findings, the clinical information, and the other modality's report. In two patients, the MRU report differed from the CTU report. CTU showed a PUJ obstruction in one and a megaureter in the other, whereas MRU showed hydronephrosis without a cause or level of obstruction. A third radiologist reviewed only the MRU images of these patients, without the CTU findings, and gave the final MRU interpretation. This adjudicated MRU interpretation was used in all analyses. The surgeons were not blinded and knew the clinical details and the ultrasound, CTU, and MRU findings.

Definition of concordance

We considered an imaging finding concordant when both the cause and anatomical level of obstruction reported on CTU or MRU corresponded to the reference-standard diagnosis. A calculus was counted as identified if seen directly or inferred from secondary signs. We considered any discrepancy between the imaging interpretation and the reference-standard diagnosis discordant. When the modality demonstrated obstruction but did not specify its underlying cause, we categorized this as "cause not correctly identified" rather than a correct identification.

Statistical analysis

Categorical variables were expressed as counts and percentages, with exact (Clopper-Pearson) 95% CIs. Because the same patients had both examinations, the two modalities represent paired, within-patient measurements; we used McNemar's exact test only as a supplementary check. With so few discordant pairs, it has very little power, so its p-value should not be read as showing or excluding a difference between the two modalities. Statistical analysis was performed using IBM SPSS Statistics, version 27.0 (IBM Corp., Armonk, NY, USA). Because all enrolled patients had established obstructive pathology and no non-obstructed comparator group was included, the correct identification rate of obstruction was estimated within the study cohort, whereas specificity, predictive values, and overall diagnostic accuracy for the presence of obstruction were not estimable.

## Results

This study was carried out at King George Hospital, Visakhapatnam. Thirty patients with suspected obstructive uropathy were enrolled after being counselled about the study protocol, and each underwent both CTU and non-contrast MRU.

Of the 48 patients assessed, 18 were excluded (MRI contraindications: 5; serum creatinine >1.5 mg/dL: 4; distal obstruction: 7; other reasons: 2, including the need for urgent decompression in 1 patient and CT performed elsewhere in 1 patient). The remaining 30 patients provided consent, underwent both CTU and MRU, and were included in the analysis (Figure 1).

**Figure 1:**
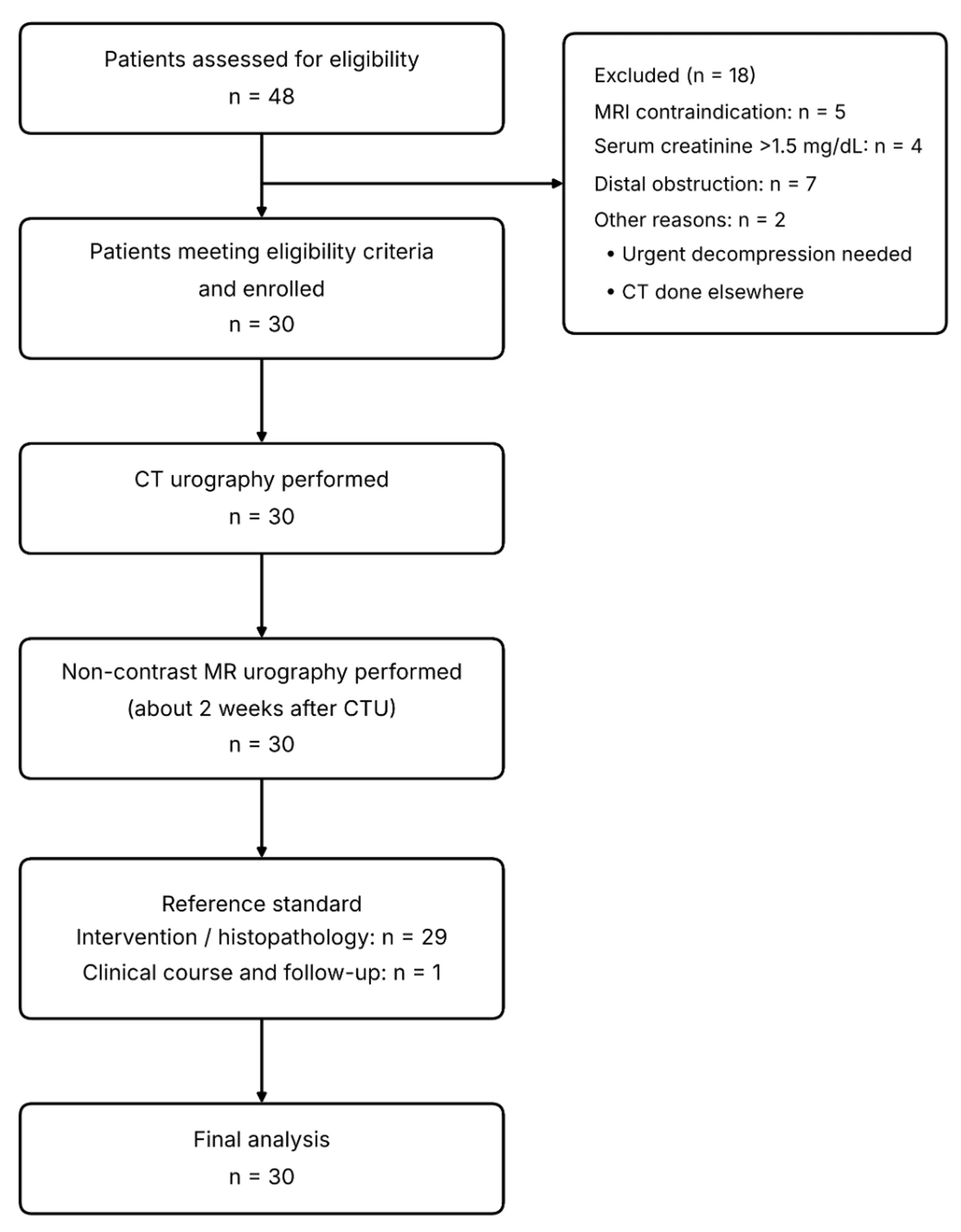
STARD 2015 flow diagram of patient selection and analysis. STARD: Standards for Reporting Diagnostic Accuracy Studies.

Demographic and clinical profile

Patients spanned a wide age range, with the largest groups in the 10-20-year and 51-60-year age brackets (6 patients each, 20%), followed by the 41-50-year age group (5 patients, 16.67%). Of the 30 patients, 18 (60%) were male and 12 (40%) were female. All patients had serum creatinine levels below 1.5 mg/dL. Loin pain was the most common presenting complaint, reported in 18 patients (60%), followed by non-localized abdominal pain in 10 patients (33.3%) and hematuria in two patients (6.7%) (Table 1 and Figure 2).

**Table 1:** Demographic and clinical characteristics of study patients.

Category	Subgroup	Count	Percentage (%)
Age group	<10 years	3	10
	10-20 years	6	20
	21-30 years	2	6.67
	31-40 years	4	13.33
	41-50 years	5	16.67
	51-60 years	6	20
	61-70 years	2	6.67
	71-80 years	2	6.67
Sex	Male	18	60
	Female	12	40
Clinical feature	Loin pain	18	60
	Non-localized abdominal pain	10	33.33
	Hematuria	2	6.67

**Figure 2:**
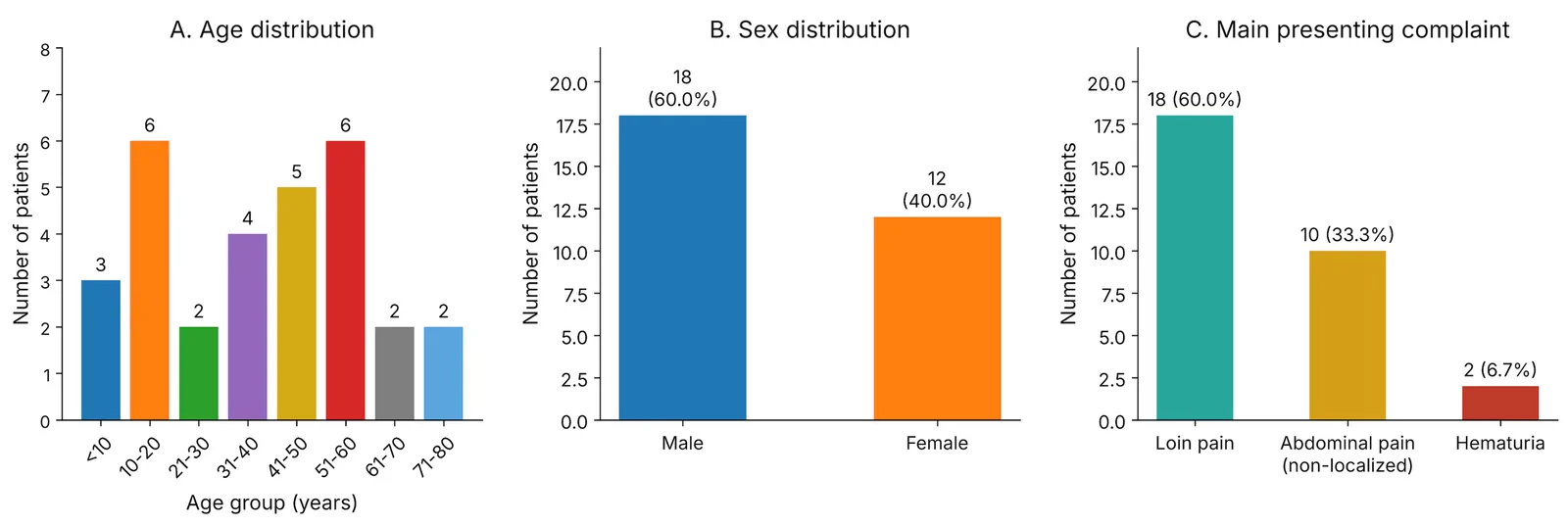
Demographic and clinical profile of study patients. Created using Matplotlib in Python 3.

One patient with a ureteric stricture had undergone ureteroscopic lithotripsy (URSL) one year before enrolment. No other patients had undergone prior stenting or surgery.

Cause of obstruction on CTU and MRU

The reference-standard diagnosis established the cause of obstruction in all 30 patients: 17 had calculus obstruction (15 with a single calculus and 2 with multiple calculi), 7 had ureteric strictures, 3 had urothelial or bladder malignancies, and 3 had congenital obstructive abnormalities (one primary obstructive megaureter and two PUJ obstruction cases). The three malignant cases comprised one bladder carcinoma and two ureteric urothelial carcinomas.

CTU correctly identified the cause in all 30 patients. Non-contrast MRU correctly identified the cause in 28 of 30 patients. MRU correctly identified all 17 calculus cases, all 7 strictures, and all 3 malignant cases. Among the three congenital obstructive abnormalities, MRU correctly identified PUJ obstruction in one patient but did not specifically identify the cause in the patient with primary obstructive megaureter or in the patient with PUJ obstruction associated with crossed fused renal ectopia. These two cases were therefore classified as "cause not correctly identified" according to the predefined criteria (Table 2, Figure 3).

**Table 2:** Cause of obstruction correctly identified on CTU and non-contrast MRU. *Includes primary obstructive megaureter (1 patient) and PUJ obstruction (2 patients). On MRU, PUJ obstruction was correctly identified in 1 of these 3 patients; the other 2 (the patient with megaureter and the patient with PUJ obstruction accompanied by crossed fused renal ectopia and an incidental large Bosniak category II cyst) were not assigned a specific cause on MRU and were counted under "Not correctly identified" for that modality. CTU: Computed tomography urography; MRU: Magnetic resonance urography; PUJ: Pelviureteric junction; TCC: Transitional cell carcinoma.

Cause of obstruction	CTU count (%)	MRU count (%)
Calculus (single)	15 (50.00)	15 (50.00)
Calculi (multiple)	2 (6.67)	2 (6.67)
Stricture	7 (23.33)	7 (23.33)
Bladder carcinoma	1 (3.33)	1 (3.33)
TCC of right ureter	1 (3.33)	1 (3.33)
TCC of left distal ureter	1 (3.33)	1 (3.33)
PUJ obstruction/megaureter (congenital obstructive abnormalities)*	3 (10.00)	1 (3.33)
Not correctly identified	0 (0.00)	2 (6.67)

**Figure 3:**
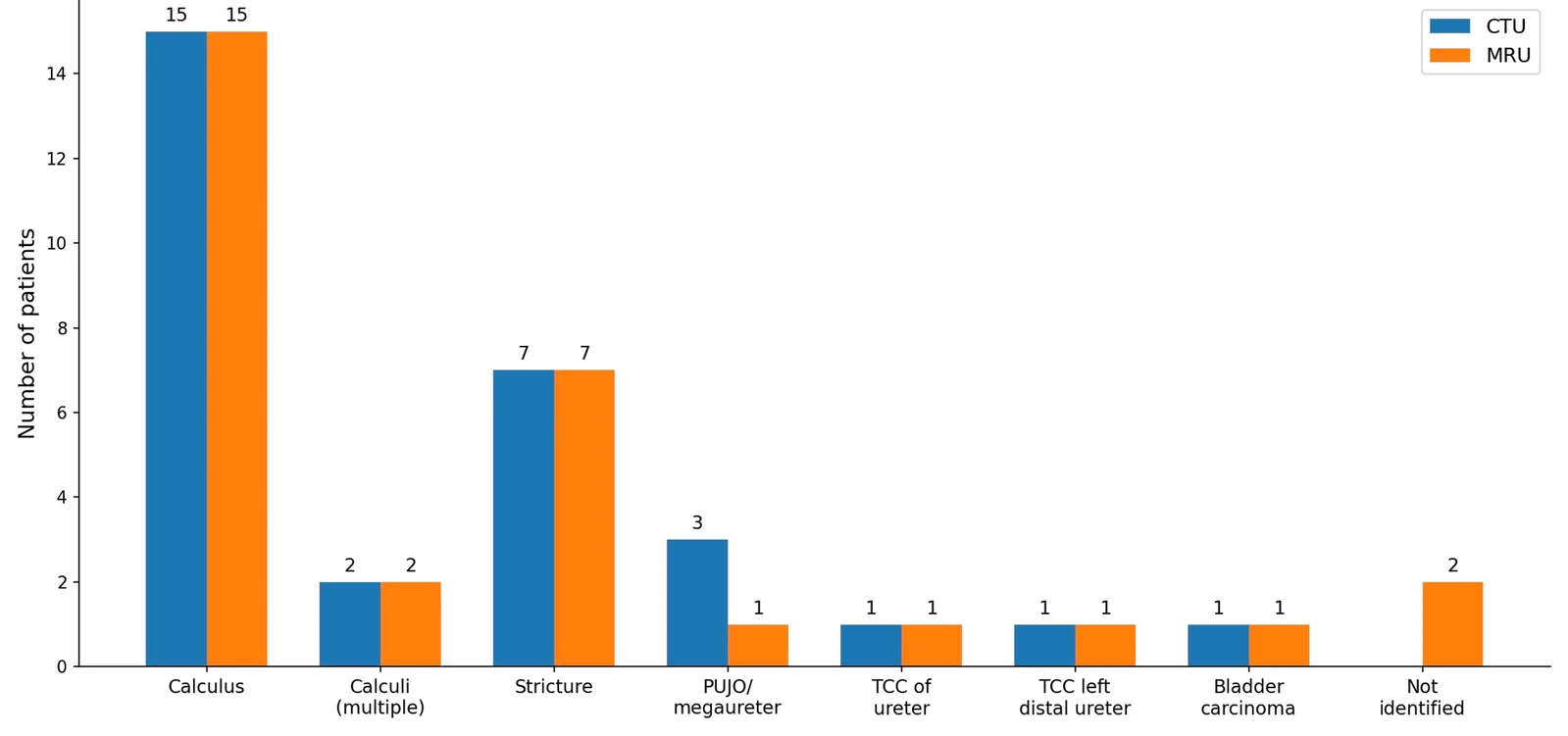
Cause of obstruction identified on CTU versus non-contrast MRU. Bars show the number of patients (out of 30) in whom each cause of obstruction was correctly identified by the respective imaging modality. CTU identified all three congenital (PUJO/megaureter) cases, whereas MRU identified only one of these three cases, with the remaining two classified as "Not correctly identified" for MRU. Created using Matplotlib in Python 3. CTU: Computed tomography urography; MRU: Magnetic resonance urography; PUJ: Pelviureteric junction; TCC: Transitional cell carcinoma.

Level of obstruction

The reference standard established the anatomical level of obstruction in all 30 patients. Against this comparator, CTU correctly localized the level in 30 of 30 patients, and non-contrast MRU in 28 of 30 patients. The two MRU failures involved the same infant with megaureter and the man with PUJ obstruction and crossed fused renal ectopia; in both cases, the dilated system was demonstrated, but the level of obstruction was not identified. After a third radiologist adjudicated these two MRU interpretations, the cause and level of obstruction remained unidentified (Table 3, Figure 4).

**Table 3:** Level of obstruction identified on CTU and non-contrast MRU. CTU: Computed tomography urography; MRU: Magnetic resonance urography.

Level of obstruction	CTU count (%)	MRU count (%)
Correctly identified	30 (100.00)	28 (93.33)
Not correctly identified	0 (0.00)	2 (6.67)

**Figure 4:**
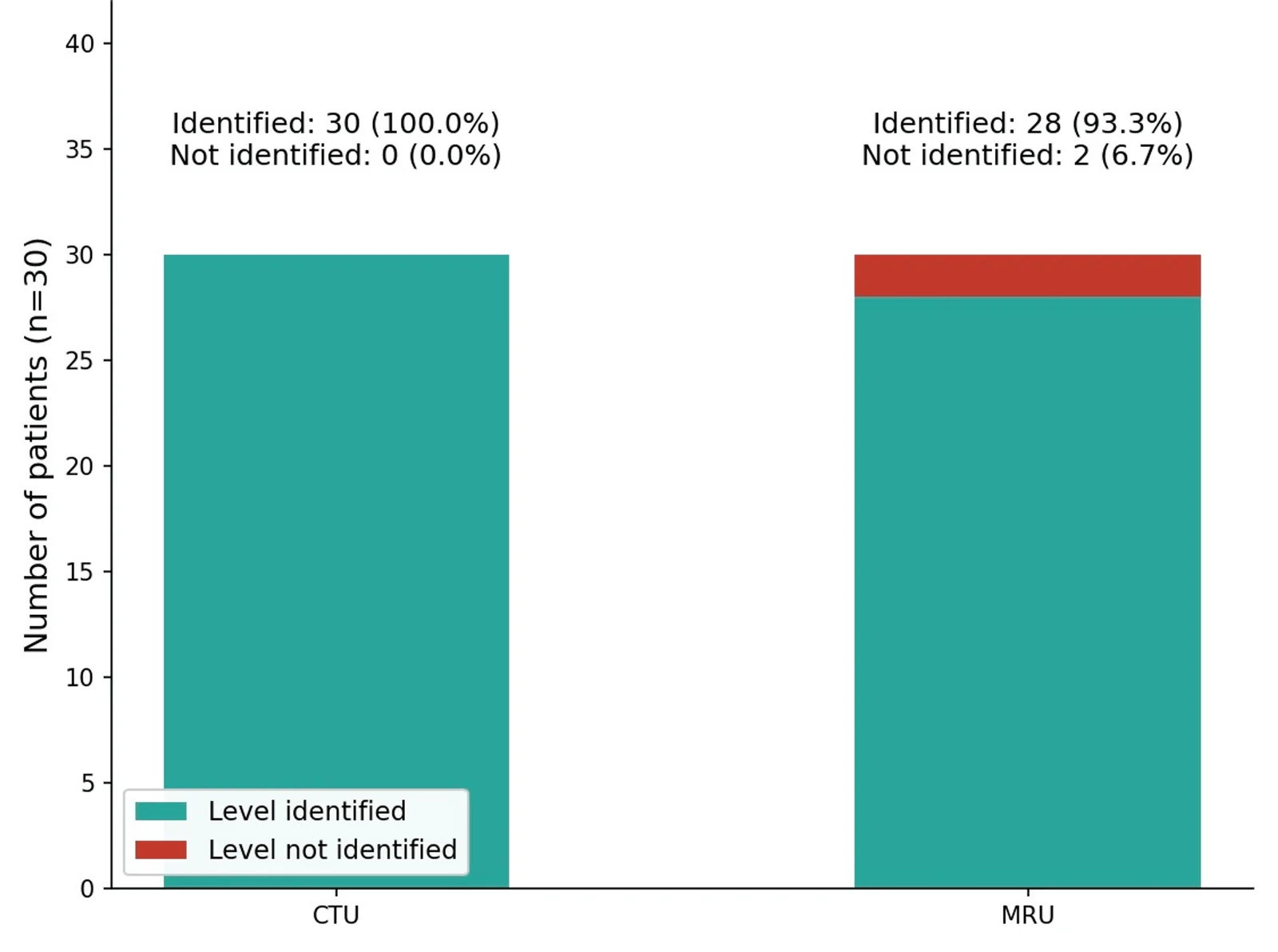
Level of obstruction correctly identified: CTU versus non-contrast MRU. Created using Matplotlib in Python 3. CTU: Computed tomography urography; MRU: Magnetic resonance urography.

Overall type of pathology responsible for obstruction

Across both modalities, calculi caused obstruction in 17 patients (56.7%), other causes (7 strictures and 3 urothelial or bladder malignancies) accounted for 10 patients (33.3%), and congenital PUJ obstruction or megaureter accounted for 3 patients (10.0%) (Table 4, Figure 5).

**Table 4:** Overall type of pathology responsible for obstruction. PUJ: Pelviureteric junction.

Type	Count	Percentage (%)
Calculus	17	56.67
PUJ obstruction/megaureter (congenital)	3	10
Others	10	33.33

**Figure 5:**
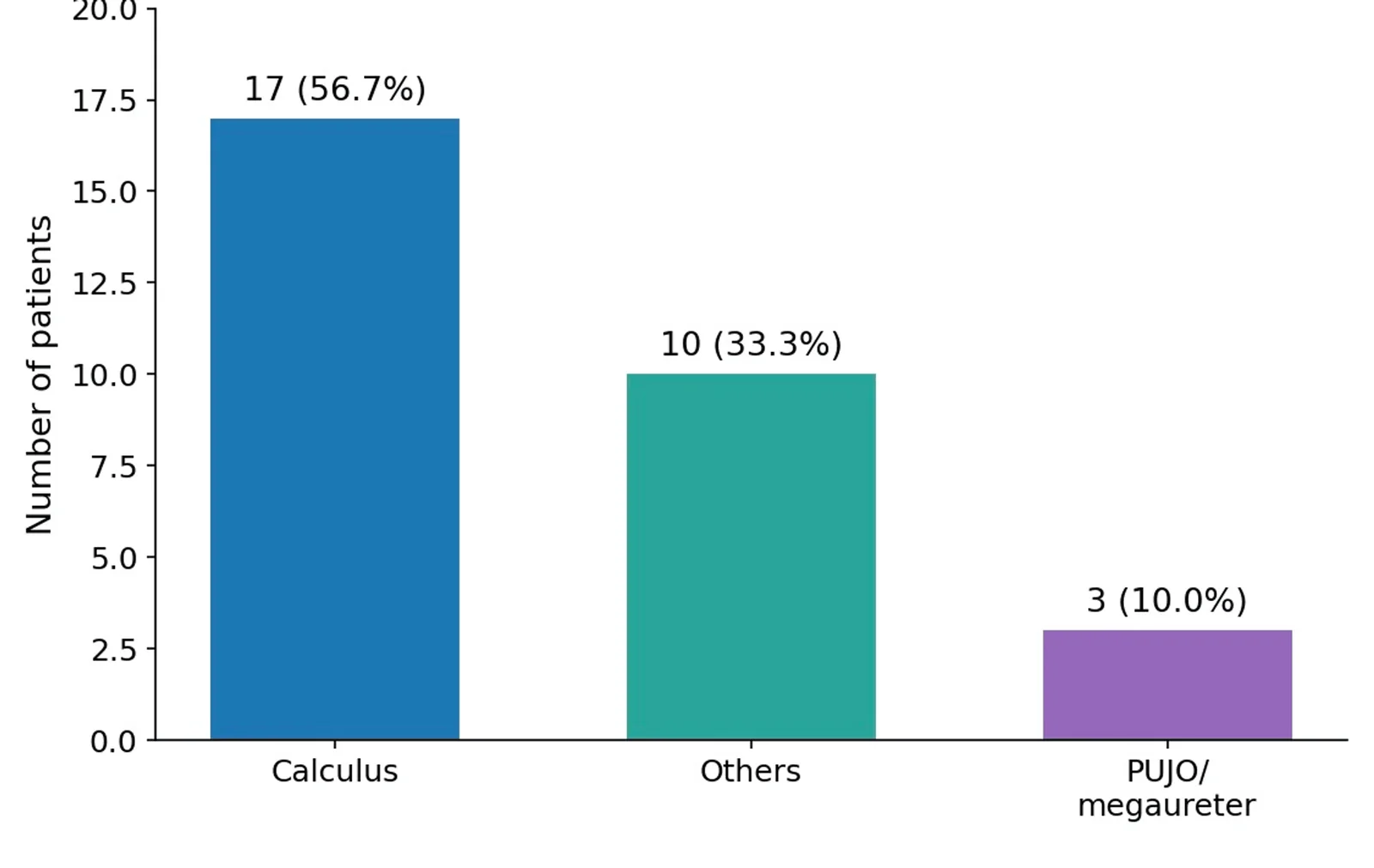
Overall type of pathology responsible for obstruction. Created using Matplotlib in Python 3. PUJO: Pelviureteric junction obstruction.

Comparative summary of CTU and MRU

Table 5 summarizes the key comparative findings from Tables 2-4 for quick reference.

**Table 5:** Comparative observed findings of CTU and non-contrast MRU. ᵃ 95% CIs were calculated using the exact Clopper-Pearson method. ᵇ Supplementary McNemar's exact test: two discordant pairs, both favouring CTU; p = 0.50. The test had low statistical power. CTU: Computed tomography urography; MRU: Magnetic resonance urography; PUJ: Pelviureteric junction.

Parameter	Confirmed by reference standard, n	CTU, n/N; correct identification rate, % (95% CI)ᵃ	MRU, n/N; correct identification rate, % (95% CI)ᵃ
Cause of obstruction (all patients)ᵇ	30	30/30; 100 (88.4-100)	28/30; 93.3 (77.9-99.2)
Level of obstruction (all patients)ᵇ	30	30/30; 100 (88.4-100)	28/30; 93.3 (77.9-99.2)
Calculus or calculi	17	17/17; 100 (80.5-100)	17/17; 100 (80.5-100)
Stricture	7	7/7; 100 (59.0-100)	7/7; 100 (59.0-100)
Urothelial or bladder malignancy	3	3/3; 100 (29.2-100)	3/3; 100 (29.2-100)
PUJ obstruction/megaureter (congenital obstructive abnormalities)	3	3/3; 100 (29.2-100)	1/3; 33.3 (0.8-90.6)
Non-congenital causes combined	27	27/27; 100 (87.2-100)	27/27; 100 (87.2-100)
Ionising radiation	-	Yes	No
Contrast agent required	-	Yes (iodinated)	No (non-contrast protocol)
Approximate acquisition time	-	12-15 min	Approximately 25-30 min

McNemar's exact test (2 discordant pairs, p = 0.50) is presented in Table 6. 

**Table 6:** Paired comparison of CTU and non-contrast MRU according to correct identification. Values represent the number of paired patients. "Correct" indicates concordance of the CTU or MRU findings with the definitive reference-standard diagnosis. McNemar's exact two-sided test was used because the observations were paired. Degrees of freedom are not applicable to the exact McNemar test. CTU: Computed tomography urography; MRU: Magnetic resonance urography.

Outcome	CTU correct / MRU correct	CTU correct / MRU incorrect	CTU incorrect / MRU correct	CTU incorrect / MRU incorrect	Total	McNemar's exact two-sided p-value
Cause of obstruction	28	2	0	0	30	0.5
Level of obstruction	28	2	0	0	30	0.5

## Discussion

This prospective paired study compared CTU and non-contrast MRU for the correct identification of the cause and anatomical level of urinary tract obstruction. Both techniques localized the obstruction well; CTU correctly identified the level of obstruction in all 30 patients, whereas MRU correctly identified the level in 28 patients (Table 5). These findings are consistent with what is already known about CTU as a widely used imaging modality for obstructive uropathy, particularly for calculi (on the unenhanced phase), strictures, and urothelial tumors (on the enhanced phases) [2,5,6]. In our series, CTU detected urinary calculi in 17 patients (56.7%), strictures in seven (23.3%), and urothelial/bladder malignancies in three (10%), consistent with its known ability to detect small, dense lesions [2,5].

MRU also performed well overall, correctly identifying the cause of obstruction in 28 of 30 patients (93.3%), including all calculi, strictures, and tumors identified by CTU. It demonstrated the dilated urinary tract, strictures, and soft-tissue lesions well [8,9]. The two cases it failed to identify correctly involved congenital obstructive abnormalities: of the three patients with PUJ obstruction or megaureter, MRU correctly identified the cause in one. In the infant with megaureter, MRU reported only hydronephrosis of unknown cause, and in the patient with PUJ obstruction and crossed fused renal ectopia, it demonstrated the ectopia and hydronephrosis but did not identify the obstruction. A possible explanation is that these conditions involve impaired drainage, which a static T2-weighted scan without contrast does not demonstrate well, whereas the excretory and delayed phases of CTU can demonstrate impaired contrast passage. This was not tested in the present study. With only three such cases, the finding is exploratory.

Urinary calculi were the most common cause of obstruction overall (56.7%), followed by strictures (23.3%). In this selected cohort of patients with established obstructive pathology, both modalities identified all 17 cases of calculous obstruction. This finding should not be interpreted as demonstrating equivalent performance for urinary calculi because the study was not designed to evaluate small, non-obstructing, or asymptomatic calculi. In addition, secondary signs of obstruction may facilitate the identification of a calculus on MRU even when the calculus itself is not directly visualized.

MRU identified all three urothelial/bladder malignancies identified by CTU in this cohort. One case of bladder carcinoma and two cases of ureteric transitional cell carcinoma (TCC) were correctly identified by both modalities. This is consistent with earlier work showing that MRU performs well for soft-tissue pathology and urothelial/bladder malignancies, particularly when gadolinium-enhanced sequences are added [10,12]. The small number of malignant cases precludes meaningful conclusions regarding comparative performance for urothelial/bladder malignancies.

MRU localized the level of obstruction in 93.3% of cases, compared with 100% for CTU, which is consistent with reports on MRU for localizing obstruction [8,9]. A recognized advantage of MRU is that it does not involve ionizing radiation, making it potentially advantageous in clinical situations where avoidance of ionizing radiation or iodinated contrast is desirable. It also allows the renal parenchyma, perirenal space, and collecting system to be assessed together in a single examination [8].

MRU also has limitations. It takes longer, costs more, and is prone to motion artifacts. It may identify a calculus only through secondary signs of obstruction without showing the stone itself. It also requires a cooperative patient and may be contraindicated in patients with certain implants (such as some pacemakers) or severe claustrophobia [10,11].

CTU correctly identified all three congenital obstructive abnormalities, whereas non-contrast MRU identified one. With so few cases, this finding is exploratory, and the choice between the two modalities should depend on the clinical situation and the patient.

Strengths

This study has several strengths. First, its prospective paired design allowed direct within-patient comparison of CTU and non-contrast MRU, minimizing between-patient variability. Second, all patients underwent both imaging modalities, allowing assessment of the same underlying pathology with each technique. Third, the examinations were interpreted by radiologists who were blinded to the ultrasound findings and clinical information, which reduces interpretation bias. Fourth, we assessed imaging findings against intervention-based or histopathological reference standards, where applicable, and used clinical and imaging follow-up in one patient. Fifth, a standardized non-contrast MRU protocol incorporating multiple sequences and 3D MIP reconstruction was used. Finally, the study included a range of clinically relevant obstructive etiologies, including calculi, strictures, congenital abnormalities, and malignancies, and evaluated both the cause and anatomical level of obstruction.

Limitations

This was a single-centre study of 30 patients with no a priori sample-size calculation, so statistical power was limited. With only two discordant pairs, McNemar's exact test had very little power, so it could neither demonstrate nor exclude a difference between the two modalities. The cohort was selected, and patients who needed urgent decompression, had undergone CT elsewhere, had an MRI contraindication, or had serum creatinine levels above 1.5 mg/dL were excluded. Selection and spectrum bias are therefore possible, and non-contrast MRU could not be assessed in patients with renal impairment. Because all patients had confirmed obstruction, only correct identification rates could be reported, not specificity or predictive values. CTU and MRU were performed within two weeks of each other, and intervention followed within one week of MRU, so stone movement or decompression could have affected the comparison, although neither was observed. The surgeons knew the clinical details and the ultrasound, CTU, and MRU findings, so incorporation bias is possible. One patient with ureteric stricture, who had undergone prior ureteroscopy, was diagnosed based on the clinical course, repeat ultrasonography, and at least 12 months of follow-up, rather than surgery or histopathology. Each modality was interpreted by one radiologist, so interobserver agreement could not be assessed, and reader experience could not be separated from the modality. Only MRU discrepancies were adjudicated. Static MRU cannot confirm functional obstruction, and diuretic renography, performed only in the congenital cases as part of routine care, was not compared with CTU or MRU. The congenital obstructive abnormalities subgroup (n = 3) is exploratory. Larger multicentre studies are needed.

## Conclusions

In this small prospective paired cohort, non-contrast MRU correctly identified the cause and anatomical level of urinary tract obstruction in 28 of 30 patients, compared with 30 of 30 patients for CTU. The two MRU failures involved congenital obstructive abnormalities: primary obstructive megaureter and PUJ obstruction associated with crossed fused renal ectopia. The study was descriptive and hypothesis-generating and was not designed to establish agreement, equivalence, or non-inferiority between CTU and non-contrast MRU. Non-contrast MRU may be an alternative in appropriately selected patients for whom avoiding ionizing radiation or iodinated contrast is desirable. Larger multicentre studies with standardized protocols, blinded interpretation, and independent reference standards are needed.
